# Response to evaluation of the food composition tables: Beyond the divergence and agreement of intakes

**DOI:** 10.1111/mcn.13195

**Published:** 2021-05-04

**Authors:** Md Ruhul Amin, Masum Ali

**Affiliations:** ^1^ Institute of Nutrition and Food Science (INFS) University of Dhaka Dhaka Bangladesh; ^2^ Department of Food Business and Development University College Cork Cork Ireland

Dear Editor,

Although we appreciate the author's attention to our paper (Ali & Amin, 2020), in our study, we did not evaluate the food composition tables (FCTs) and their methodological development as they imply. Instead, we examined the comparability and the agreements of the estimated nutrient intakes between the two FCTs of Bangladesh. This type of research is undertaken when different FCTs are used simultaneously for the same population (Deharveng et al., 1999; Garcia et al., 2004; Hakala et al., 2003). We have found that studies by Rahman et al. (2016) and Rahman et al. (2017) used FCTB‐2012, though FCTB‐2013 was available at that time and was concomitantly used by the other researchers (Al Hasan et al., 2016; Campbell et al., 2018). Thus, the estimated nutrient intakes in these studies have been threatened by concerns about reliability.

The authors are correct that de‐attenuated correlation may improve the energy‐adjusted agreement, but this was beyond our study methodology. However, the authors misinterpreted our study findings. The authors selectively picked up the weighted kappa value of ‘poor’ for four nutrients but ignored the ‘slight’ and ‘fair’ categories of agreements for other micronutrients (Landis & Koch, 1977). We included 15 micronutrients that are matched between the two FCTs, which have significant public health and clinical implications as FCTs are widely used for the development of dietary guidelines and modification of diets for the treatment of diseases (ICMR‐NIN, 2020). Although we stand behind our methodological approach used in our study, we performed the Bland–Altman test (Bland & Altman, 2010) for the additional insights (bias) as per query. This analysis shows that vitamin C, beta‐carotene, vitamin B6, folate, zinc and potassium are highly biased (an indication of disagreement) towards the FCTB‐2013, while thiamine, riboflavin, iron, calcium, and sodium are biased towards the FCTB‐2012 with a wider range of limit of agreements (LOA) (Table 1). Thus, our original conclusions are correct and based on evidence that estimated micronutrients from FCTs should be interpreted cautiously.

**TABLE 1:** Level of agreement for intakes of energy, macro‐ and micronutrients between FCTB‐2012 and FCTB‐2013

Nutrients	Mean difference^a^ (95% CI)	*p* value	Mean^b^	% Bias^c^	LOA (reference range for difference)
Energy (kcal)	17.54 (6.07–29.0)	0.003	2884.98	1	−52.72 to 87.79
Protein (g)	−4.06(−4.93 to −3.19)	<0.001	72.38	−6	−9.38 to 1.26
Fat (g)	0.88 (0.45–1.32)	<0.001	29.38	3	−1.79 to 3.56
Carbohydrate (g)	26.17 (22.14–30.19)	<0.001	570.66	5	1.50 to 50.83
Vitamin C (mg)	−113.84 (−148.3 to −79.32)	<0.001	125.97	−90	−325.33 to 97.66
Beta‐carotene (μg)	−3356.65 (−4215.52 to −2497.78)	<0.001	2443.03	−137	−8620.26 to 1906.95
Thiamine (mg)	0.26 (0.21–0.31)	<0.001	1.39	19	−0.05 to 0.57
Riboflavin (mg)	0.24 (0.18–0.29)	<0.001	0.96	25	−0.09 to 0.56
Niacin (mg)	0.65 (−1.20 to 2.49)	0.48	23.67	3	−10.69 to 11.99
Vitamin B6 (mg)	−1.06 (−1.21 to −0.90)	<0.001	1.20	−88	−2.02 to −0.10
Folate (μg)	−131.51(−161.42 to −101.59)	<0.001	150.29	−88	−314.84 to 51.82
Copper (mg)	1.18 (0.82–1.53)	<0.001	4.34	27	−0.99 to 3.34
Zinc (mg)	−7.13 (−7.76 to −6.48)	<0.001	10.84	−66	−11.03 to −3.22
Iron (mg)	9.70 (7.50–11.88)	<0.001	19.63	49	−3.74 to 23.13
Calcium (mg)	58.38 (43.82–72.93)	<0.001	311.38	19	−30.82 to 147.59
Magnesium (mg)	109.25 (54.53–163.97)	<0.001	622.62	18	−226.11 to 444.61
Sodium (mg)	41.00 (13.07–68.92)	0.005	197.18	21	−130.14 to 212.14
Potassium (mg)	−412.51 (−525.76 to −299.25)	<0.001	2026.05	−20	−1106.60 to 281.57
Phosphorus (mg)	11.60 (−18.41 to 41.61)	0.43	1313.96	1	−172.34 to 195.54

Abbreviations: CI, confidence interval; LOA, limit of agreement.

^a^ Mean difference = FCTB‐2012 − FCTB‐2013.

^b^ Mean = mean of both FCTB.

^c^ % Bias = mean differences divided by the mean value.

## CONFLICTS OF INTEREST

The authors declare that they have no conflicts of interest.
